# Illustrated key to the adult female *Anopheles* (Diptera: Culicidae) mosquitoes of Sri Lanka

**DOI:** 10.1007/s13355-016-0455-y

**Published:** 2016-12-28

**Authors:** Nayana Gunathilaka

**Affiliations:** Department of Parasitology, Faculty of Medicine, University of Kelaniya, Ragama, Sri Lanka

**Keywords:** *Anopheles*, Vector

## Abstract

The identification of adult female anopheline mosquitoes is an important aspect in malaria surveillance and control strategy throughout the world, and taxonomic keys are being regularly revised and updated as new information becomes available. However, the currently available key to the anophelines of Sri Lanka is of limited use, because they were published more than 25 years ago. This paper presents an illustrated key for the identification of 23 adult female *Anopheles* mosquitoes which are currently recognized as local anopheline species in Sri Lanka.

## Introduction

The genus *Anopheles* is the only mosquito taxon known to transmit human malarial protozoa. Anophelines are also known to be capable of transmitting dirofilarial nematodes and arboviruses of veterinary and medical importance (Ramachandra 1984). The taxonomy of this group of mosquitoes is, therefore, of great importance, since vector incrimination is dependent upon accurate species identification. Traditional field taxonomy based on morphological characteristics remains the backbone of all vector control programs. Therefore, there is a need for continual revision and improvement of morphological keys for the identification of members of this important group of insects (Amarasinghe 1990).

Previous records of Sri Lankan *Anopheles* were imperfect, and even the number and names of the species present were doubtful. An attempt to remedy the matter was made by Dr. A. J. Chalmers in 1905. Chalmers presented the results of investigations made in various parts of the island during the dry season, and also incorporated the records of previous observers. The species list was revised by Carter (1950) and subsequently by Jayasekara and Chelliah (1981). Twenty-two anopheline species were recorded in Sri Lanka by Amarasinghe (1990).

Two illustrated keys to the *Anopheles* of Sri Lanka (Amarasinghe 1990; Carter 1950) are of limited value, as these were published more than 25 years ago and significant advances in our knowledge of the *Anopheles* mosquitoes have occurred in the intervening years. The purpose of the key presented herein is to assist entomologists in identifying adult female *Anopheles* mosquitoes. The key can be used to initially allocate specimens to species group and then to species.

## Materials and methods

The morphological characteristics used here were based on original observations and previous usage in the literature. The following publications were consulted during the construction of this key (Amarasinghe 1990; Christophers 1933; Colless 1957; Harrison 1980; Harrison and Scanlon 1975; Reid 1968; Rattanarithikul et al. 2006; Sallum et al. 2005).

Taxonomic characteristics were checked against Sri Lankan specimens by examining the persevered reference specimens archived at the Faculty of Medicine, University of Kelaniya, Sri Lanka.

Generally, two or more primary characteristics are used in each step in the key, with the intension of making them user-friendly for field taxonomists. Species nomenclature follows that proposed by Knight and Stone (1977), and abbreviations used in the text follow those used by Reinert (1975, 2001). Nomenclature for morphological characteristics follows Harbach and Knight (1980, 1982).

The key was distributed to the 17 field entomological surveillance teams in Mannar, Trincomalee, Ampara, Batticaloa, and Killinochchi Districts attached to the Tropical and Environmental Diseases and Health Associates (TEDHA) malaria elimination program and to some selected entomological teams in the Anti Malaria Campaign (AMC).

Reported species were identified using the key for an 8-month period, and feedback was obtained from the field entomological teams. The key was validated on the basis of the feedback. In case of doubt, it is essential to consult published literature with detailed descriptions of species.

## Results

**Fig. 1:**
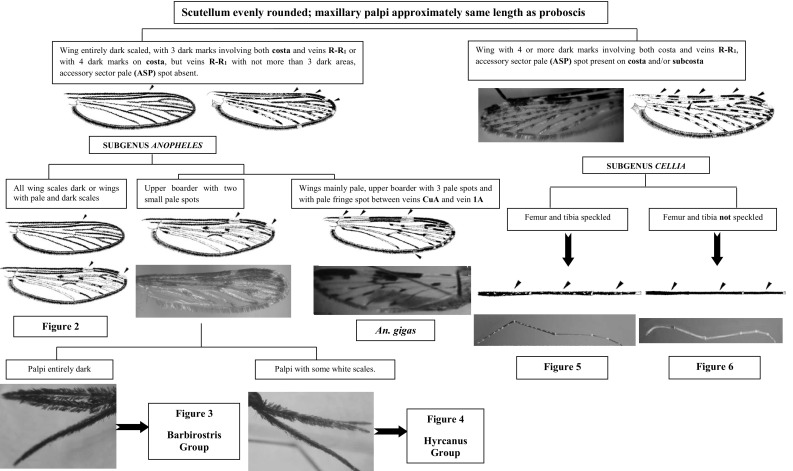
Key to the adult female anophelines in Sri Lanka

**Fig. 2:**
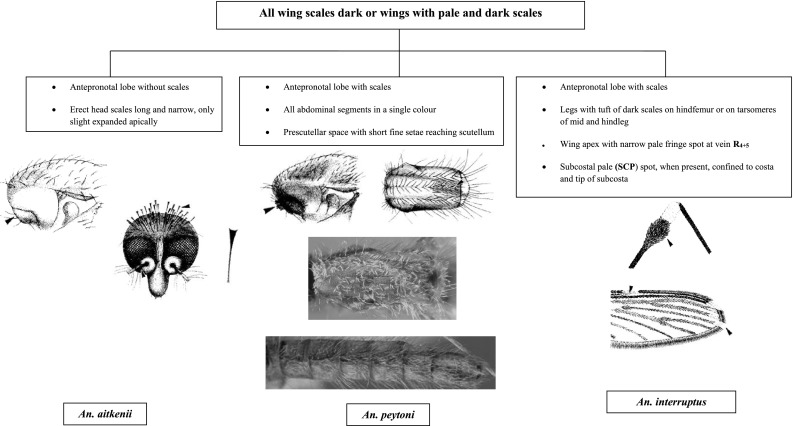
Mosquitoes under subgenus *Anopheles* with all wing * scales dark* or wings with * pale * and * dark scales*

**Fig. 3:**
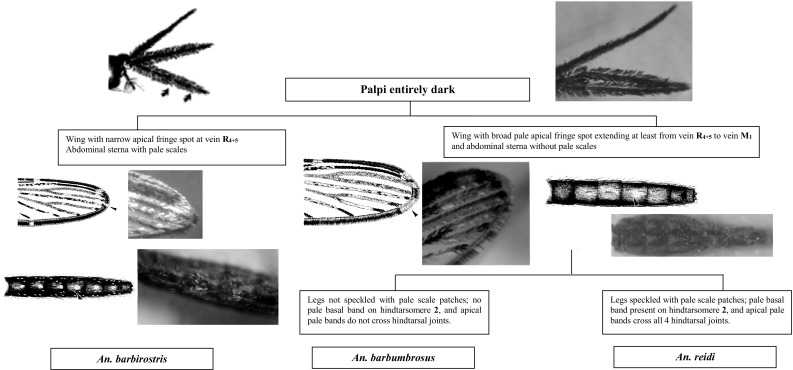
Members in the Barbirostris group

**Fig. 4:**
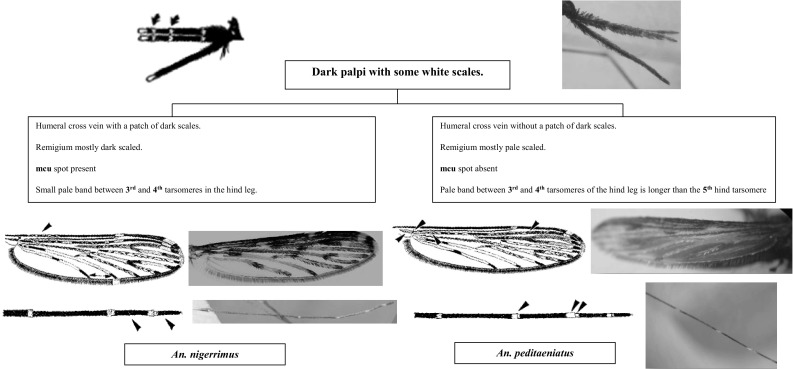
Members in the Hycanus group

**Fig. 5:**
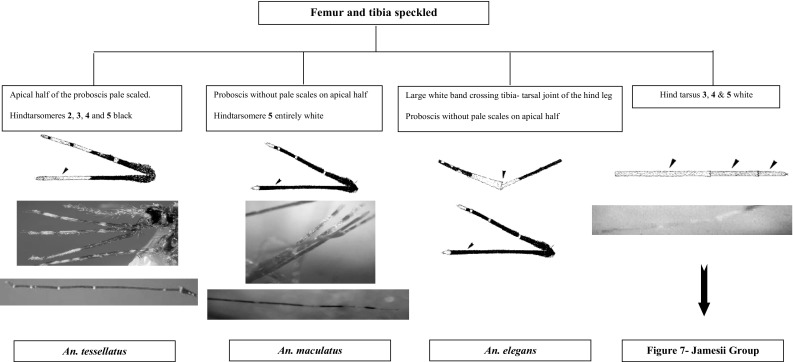
Members in the Subgenus *Cellia* with fumur and tibia speckled

**Fig. 6:**
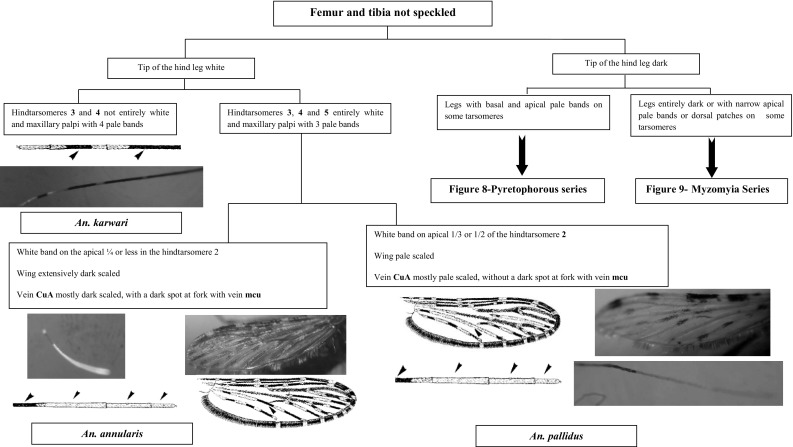
Members in the Subgenus *Cellia* with fumur and tibia not speckled

**Fig. 7:**
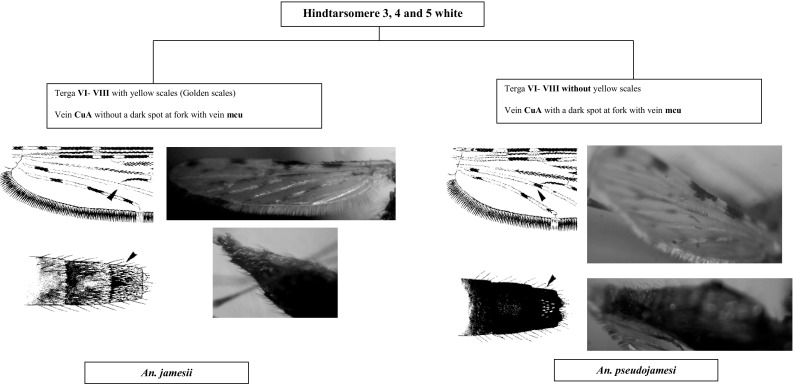
Mosquitoes under Jamesii group

**Fig. 8:**
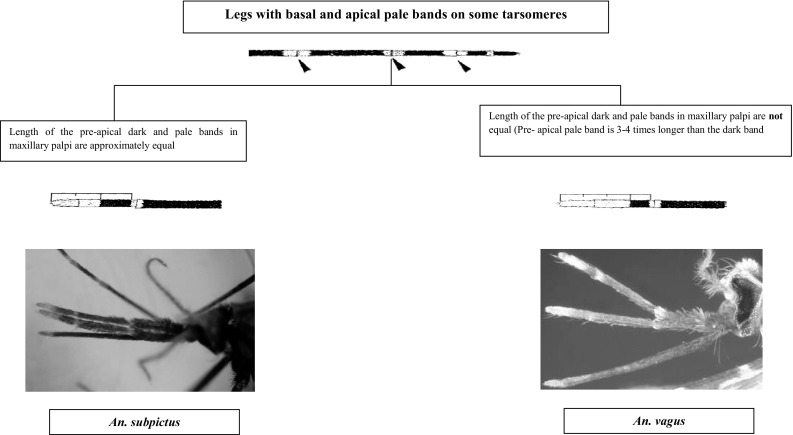
Anophelines under Pyretophorous series

**Fig. 9:**
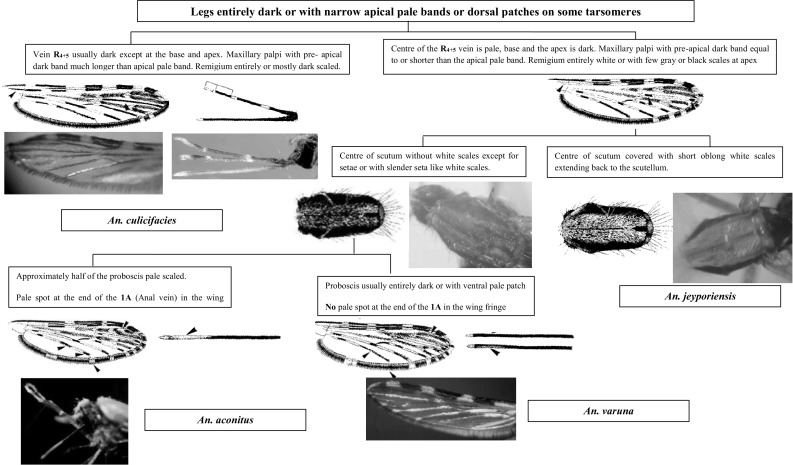
*Anopheles* species under Myzomyia series

This section presents an illustrated key for the identification of 23 adult female *Anopheles* mosquitoes which are currently recognized as the local anopheline species in Sri Lanka. Morphological features of *An. peytoni* and *An. jeyporiensis* have been included in addition to the anopheline keys published previously. The revised adult morphological key is shown below (Figs. 1, 2, 3, 4, 5, 6, 7, 8, 9). The species included in the key are as follows:

**Subgenus** ***Anopheles***: *aitkenii* James, *barbirostris* Van der Wulp, *barbumbrosus* Strickland and Choudhury, *gigas refutans* Alcock, *interruptus* Puri, *nigerrimus* Giles, *peditaeniatus* (Leicester), *peytoni* Kulasekera, Harrison and Amerasinghe, *reidi* Harrison.

**Subgenus** ***Cellia***: *aconitus* Donitz, *annularis* Van der Wulp, *culicifacies* Giles, *elegans* (James), *jamesii* Theobald, *jeyporiensis* James, *karwari* (James), *maculatus* Theobald, *pallidus* Theobald, *pseudojamesi* Strickland and Chowdhury, *subpictus* Grassi, *tessellatus* Theobald, *vagus* Donitz, *varuna* Iyengar.

## Discussion

This key is meant as an aid to the rapid identification of anophelines in Sri Lanka. It has thus been made as simple and concise as possible, using a few recognized primary characteristics at each step. Steps that would assign various species to their respective series and species groups are included wherever essential.

The illustrated key includes 23 *Anopheles* species which are currently known to occur in Sri Lanka. *Anopheles peytoni* and *An. jeyporiensis*, which were not included in previous keys for the anophelines of Sri Lanka, are included here.

Subgenus *Anopheles*: The following morphological features distinguish species of this subgenus from those of the subgenus *Cellia*: wings entirely dark-scaled or with three dark marks involving the costa and veins R–R_1_. The Sri Lankan anophelines of this subgenus belong to three taxonomic series: the Myzorhynchus, Lophoscelomyia, and Anopheles series.

Species of the Myzorhynchus series can be separated from species of the other series in Sri Lanka by following characteristics: (i) wing with dark and pale scales, upper boarder with two small pale spots; (ii) antepronotum with scales; (iii) maxillary palp with dark and erect scales (shaggy appearance); (iv) basal third of fore femur swollen; (v) hind femur without a distal broad white band; (vi) head scales always broad; (vii) coxae often with scales; and (viii) tarsi with pale bands.

Species of the Hyrcanus and Barbirostris groups of the Myzorhynchus series are present in Sri Lanka. These two groups can be separated easily by the ornamentation of the maxillary palpus, which is entirely dark-scaled in species of the Barbirostris group and has some white scales in species of the Hyrcanus group.

The Hyrcanus group includes two species in Sri Lanka, *An. nigerrimus* and *An. peditaeniatus*. There is a considerable degree of confusion regarding their identification. The most reliable identification features are included in the key. The pale scaling on the remigium is particularly prominent in Sri Lankan *An. peditaeniatus* (Amarasinghe 1990). Most specimens have a silvery white appearance (Reid 1953). Also the mediocubital crossvein (mcu) is entirely dark scaled. *Anopheles nigerrimus* seems to be a dark species when compared with *An. peditaeniatus*. It has dark scales on the humeral crossvein, the remigium has dark scales, the mcu has pale scales, and the apical dark mark on the anterior cubitus (CuA) is short, rarely as long as the apical dark mark on the anal vein (1A). The presence of a pale band between hindtarsomeres 3 and 4 is not reliable and has probably caused misidentifications by the field staff. Harrison and Scanlon (1975) recommended that the use of the hindtarsal bands as a key characteristic of *An. peditaeniatus* should be discontinued. However, continuing confusion regarding the identification of the two species could adversely impact on current malaria elimination programs in Sri Lanka, as *An. nigerrimus* is considered as a potential vector for malaria transmission (Amarasinghe 1990; Gunathilaka et al. 2014, 2016) whereas *An. peditaeniatu*s is not a vector.

Members of the Barbirostris group are another difficult group with regard to the separation of adults on the basis of morphological features. Of the three species present in Sri Lanka, *An. reidi* can be distinguished from *An. barbirostris* and *An. barbumbrosus* by the presence of speckled legs, a pale basal band on hindtarsomere 2, and pale bands crossing all four of the hind tarsal joints (Harrison, 1973).

Adults of both *An. barbirostris* and *An. barbumbrosus* have characteristics that differ from the species from Southeast Asia, but these differences apparently reflect intraspecific geographical variations. Adult *An. barbirostris* in the country typically have dark wings with a narrow apical fringe spot at the vein R_4+5_ and numerous pale scales on the abdominal sterna, characteristics that are usually found on *An. campestris* Reid, in Malaysia and Thailand. According to some studies conducted by Amarasinghe (1990) in the North Central and Eastern Provinces in Sri Lanka have found some *An. barbirostris* specimens with similar morphological features to *An. campestris*. However, the present study did not find any species with similar morphological features to *An. campestris*.

Some recent studies conducted in Sri Lanka has characterized *An. barbirostris* using mtDNA cytochrome oxidase subunit I (COI) and ribosomal RNA internal transcribed spacer 2 (ITS2) gene sequences. These studies have assumed that the specimens collected from widely separated locations in Sri Lanka with morphology characteristics of *An. barbirostris s.l.* form a new molecular type with close resemblance to *An. barbirostris s.s* from Indonesia and Thailand (Gajapathy et al. 2014).

Adult *An. barbumbrosus* in Sri Lanka has the wing with an accessory apical pale fringe spot at vein R_4+5_ to M_1+2_ and abdominal sterna without pale scales which are similar in morphological features to *An. reidi*. Adults of the Barbirostris group obtained during field collections always suffer varying degrees of damage to the wing fringe. Hence, the identification on the basis of the fringe spots is difficult or impossible. Therefore, the abdominal and limb characteristics are thus the most workable for routine basis.

Species of the Lophoscelomyia series can be distinguished from the other Sri Lankan members of subgenus *Anopheles* by the following combination of characteristics: (i) wing with dark and pale scales; (ii) antepronotum with scales; (iii) apex of hind femur with prominent tufts of erect black and white scales; (iv) head with erect broad scales on vertex; (v) scutum pale centrally and dark laterally, pleura dark; (vi) coxae pale, fore and mid coxae with long, curved scales projecting posterior-ventrally; (vii) tarsomeres without pale bands; and (viii) abdominal sternum VIII with scales (Harrison and Scanlon, 1975). A single species, *An. interruptus*, belonging to the Asiaticus group is present in Sri Lanka.

The Anopheles series is considered as an extremely variable series and thus difficult to define; most species in this series cannot be reliably separated on the basis of adult female morphology (Amarasinghe 1990). There are three recorded species (*An. aitkenii*, *An. peytoni*, and *An. gigas*) in Sri Lanka. *Anopheles aitkenii* can be separated from others by the absence of scales in the antepronotal lobe; long and narrow erect head scales, slightly expanded apically.

*Anopheles peytoni* is a unique and well-differentiated species that exhibits characteristics similar to several diverse members of the Aitkenii group. Generally, the adult females are mostly similar to *aitkenii*, *bengalensis*, *borneensis*, *fragilis*, *stricklandi*, and *tigertti* (Kulasekara et al. 1988). Most of the other members of the Aitkenii group have culicine like in general. The antennae of male *An. peytoni* are strongly plumose like female antennae. Otherwise, adult *An. peytoni* cannot be separated from the other Aitkenii group members except by male genitalia characteristics.

*Anopheles gigas* has narrowly banded palp and tarsi and a few scales on the coxae (Amarasinghe 1990; Reid 1968). The wing is brightly ornamented. It can be easily mistaken for that of subgenus *Cellia*, but there is no accessory sector pale (ASP) spot in costa and vein R–R_1_ lacks four dark spots, which are specific for the subgenus *Cellia*. However, this species can be separated by the presence of dark wing fringe except a pale patch between CuA.

The following morphological features distinguish species of this subgenus from those of subgenus *Anopheles*: wing with four or more dark marks involving both costa and veins R–R_1_, presence of ASP spot on cosa and/or subcosta. The Sri Lankan anophelines of this subgenus belong to four taxonomic series: the Myzomyia, Pyretophorous, Neocellia, and Neomyzomyia series.

Under the Myzomyia series there are four species in Sri Lanka (*An. aconitus*, *An. culicifacies*, *An. jeyporiensis*, and *An. varuna*). All these four species have common morphological characteristics such as propleuron with 1–4 setae, palpi with three pale bands, antepronotum without scales, legs entirely dark with narrow apical pale bands on some tarsomeres and abdominal segments VII–VIII, and female cerci without scales (Amarasinghe 1990; Reid 1968).

*Anopheles aconitus* can be recognized from other members especially by the presence of a flavescence proboscis, extensive pale scalation of wing vein R_4+5_, and wide pale apical and subapical palpal bands and pale spot at the end of the 1A in the wing fringe can be considered as secondary identification features.

*Anopheles culicifacies* has maxillary palpi with a preapical dark band much longer than the apical pale band, remigium entirely or mostly dark scaled, and vein R_4+5_ usually dark except at the base and apex.

The following characteristics are common for both *An. varuna* and *An. jeyporiensis*: (i) apical 1/2 of the proboscis is not white; (ii) no pale spot at the end of 1A in the wing fringe; and (iii) center of R_4+5_ vein is pale except the base and apex. *Anopheles jeyporiensis* is a unique and well-differentiated species that exhibits characteristics similar to several diverse members of the Myzomyia series. However, they can be separated from others by the center of the scutum with pale scales extending to scutellum and vein R1 with an accessory pale spot on the preapical dark (PD) area (Gunathilaka et al. 2013, 2015; Gunathilaka 2014). Anyhow, previous studies confirmed that this species is rather variable, especially in the wing markings. The proportion of dark and pale on the female palp is also rather variable (Christophers 1933).

Pyretophorous series can be separated from the other series of subgenus* Cellia* in Sri Lanka by the following combination of characteristics: (i) propleuron with 1–4 setae; (ii) hind tarsomere 5 at least partially dark; (iii) palps with three pale bands; (iv) antepronotum without scales; (v) fore leg with basal and apical pale bands on some tarsomeres; and (vi) abdominal segments vi–viii and female cerci with at least a few scales (Harrison 1980; Reid 1968). Two species, *An. subpictus* and *An. vagus*, occur in Sri Lanka. Apart from the key characteristic of the palpal banding pattern, there are no other reliable features for separating these two species, except possibly for the pale patch at the apex of the proboscis in *An. vagus*, which is absent in *An. subpictus* (Reid 1968). However, samples collected from coastal side of the Vankalai area in the Mannar District of Sri Lanka presented some morphological features similar to *An. sundicus*, *An. pseudosundicus*, and *An. epiropticus* (Gunathilaka et al. 2011). Therefore, more studies are essential to explore this context with the aid of molecular entomological tools.

There are six species in Sri Lanka under the Neocellia series (Annularis group: *An. annularis*, *An. pallidus*; Jamesii group: *An. jamesii*, *An. pseudojamesi*; *An*. *maculatus* and *An. karwari*). The following features can be listed as unique features for this series: (i) absence of propleural setae; (ii) hindtarsomere 5 entirely pale scaled; and (iii) mesonotum with broad pale or white scales (Amarasinghe 1990; Reid 1968). For easy identification these six species can be categorized as *An. jamesii*, *An. pseudojamesi*, and *An. maculatus* having speckled legs and *An. annularis*, *An. pallidus*, and *An. karwari* having no speckled legs. Of these, *An. karwari* and *An. maculatus* have only the last hindtarsi entirely white. However, these two species can be separated by the presence of speckled legs only in *An. maculatus*. Other members have hindtarsi 3–5 entirely white. From these *An. annularis* and *An. pallidus* can be separated by the absence of speckled legs.

The wing of *An. annularis* is extensively dark scaled and the vein CuA is mostly dark scaled, with a dark spot at the fork with the vein mcu, whereas the wing of* An. pallidus* is pale in color and the vein CuA mostly pale scaled, without a dark spot at the fork with the vein mcu. Furthermore, *An. annularis* has a white band on the apical 1/4 or less in the hindtarsomere 2, whereas* An. pallidus *has a white band on apical 1/3 or 1/2 of the hindtarsomere 2. However, the ratio of white area on the hindtarsomere 2 is variable in both species and difficult to use as the man point of differentiation.

Separation of *An. jamesii* can be done by the presence of golden color scales in the abdominal terga vi–viii and vein CuA without a dark spot at the fork with vein mcu, whereas *An. pseudojamesi* has no golden color scales in the abdominal terga vi–viii and vein CuA with a dark spot at the fork with the vein mcu.

The Neomyzomyia series includes two species, *An. tessellatus* and *An. elegans*. The wings of these two species are highly spotted on vein radius (R), media (M), CuA, and 1A. Commonly they have propleuron with 1–4 setae; antepronotum with scales; palps with four or more pale bands; hindtarsomere 5 at least partly dark scaled; legs usually speckled with pale patches; and wings with many small dark marks, usually four or more in vein 1A. (Harrison 1980; Reid 1968). In general the presence of four or more spots on vein 1A is useful to distinguish these two species from other Sri Lankan anophelines.

*Anopheles tessellatus* has a flavescence proboscis like in *An. aconitus*. This similarity means that there is a considerable chance of misidentifying the species as *An. aconitus*. Therefore, the species should be tested for speckled leg characteristics and number of wing spots for accurate identification. *Anopheles elegans* has a unique feature to distinguish it from others. It has a large white band crossing the tibia–tarsal joint of the hind leg.

Morphological identification keys prepared with digital pictures may facilitate accurate identification by field taxonomists. Key diagnostic characteristics have been highlighted with diagrams and digital photographs in order to help taxonomists recognize distinguishable morphological characteristics. This key is meant as an aid to the rapid identification of anophelines in Sri Lanka. It has thus been made as simple and concise as possible, using a few recognized primary characteristics at each step. Steps that would assign various species to their respective series and species groups are included wherever essential. Each characteristic has been described through a technical description. Hence, this study would be essential for speedy and accurate species identification in order to strengthen and support current malaria elimination programs through appropriate vector identification.

## Conclusion

The identification of adult anopheline mosquitoes is an important aspect of malaria surveillance and control programs.
